# miR-1258 Regulates Cell Proliferation and Cell Cycle to Inhibit the Progression of Breast Cancer by Targeting E2F1

**DOI:** 10.1155/2020/1480819

**Published:** 2020-07-15

**Authors:** Xianbao Zhao

**Affiliations:** Department of Oncology, Yiwu Central Hospital, No. 699 Nanmen Road, 322000 Yiwu, China

## Abstract

**Objective:**

This study is designed to clarify that miR-1258 targets E2F1 to regulate the proliferation and cell cycle of breast cancer (BC) cells and consequently suppress the progression of BC.

**Methods:**

Bioinformatics analysis was used to analyze the differentially expressed genes in BC. The expression of miR-1258 and E2F1 mRNA in BC cell lines and immortalized breast epithelial cell lines were detected by qRT-PCR. The proliferation and growth activity of BC cells were detected by MTT and colony formation assays. The apoptosis and cell cycle of BC cells were detected by flow cytometry and the targeting relationship between miR-1258 and E2F1 was identified by dual-luciferase assay.

**Results:**

The expression of miR-1258 was decreased while that of E2F1 was increased in BC cells. Overexpression of miR-1258 and silencing E2F1 could inhibit the cell proliferation and growth, block cells in the G0/G1 phase, and promote cell apoptosis. Besides, miR-1258 inhibited cell proliferation and growth, block cells in the G0/G1 phase, and promote cell apoptosis by downregulating E2F1.

**Conclusion:**

miR-1258 regulates the proliferation and cell cycle to inhibit the progression of BC by targeting and downregulating E2F1.

## 1. Introduction

Breast cancer (BC) is a hormone-dependent cancer most frequently diagnosed in women, and it poses a serious threat to women's life and health [1, 2]. There are many pathogenic factors leading to BC, including age, overweight, alcohol abuse, and smoking. Intensive studies and improved treatments have diminished the mortality of BC in recent years, but the mortality still accounts for 9.6% of global cancer-related deaths [3, 4]. Therefore, in-depth discussion on the molecular mechanism underlying BC occurrence and progression and identification of potential molecular therapeutic targets for BC are of great significance for reducing BC mortality.

MicroRNAs (miRNAs), small non-coding RNA molecules expressed in different tissue and cell types, are key regulators inhibiting the expression of target genes, and the dysregulation of miRNAs tends to initiate various diseases [5]. miR-1258 regulates the occurrence and development of multiple cancers, such as oral squamous cell carcinoma, liver cancer, and gastric cancer [5–7], and it also shows a relationship with BC to some extent with its expression lowly expressed [8]. This study examined the effect of miR-1258 overexpression on BC cells, as well as predicted and validated the target gene of miR-1258 to state the mechanism of miR-1258 regulating the progression of BC.

As a member of the E2F family, E2F1 encodes the transcription factor E2F1 protein, which plays an important role in cell proliferation and apoptosis by regulating the expression of various genes [9, 10]. In this study, bioinformatics analysis was used to predict the downstream target gene of miR-1258, finding that there was a binding site of miR-1258 on E2F1 3′UTR. Meanwhile, published literature has indicated that E2F1 is related to the prognosis of BC. The targeting relationship between miR-1258 and E2F1 was verified, and the effects of miR-1258 and E2F1 on BC cells were observed.

This article is aimed at studying the role of miR-1258 in BC and predicting its target gene to provide a theoretical basis for the diagnostic and therapeutic values of miR-1258 in BC.

## 2. Methods

### 2.1. Bioinformatics Analysis

The miRNA and mRNA expression profiles of BC were downloaded from the TCGA-BRCA dataset (https://portal.gdc.cancer.gov/), and differential analysis was conducted by edgeR package with ∣logFC | >2 and padj < 0.05 as threshold. Survival analysis of the differentially expressed miRNAs (DEmiRNAs) was conducted combined with the clinical information of the samples to determine the target miRNA. Thereafter, the target genes for the miRNA were predicted by TargetScan (http://www.targetscan.org/vert_71/), miRDB (http://www.mirdb.org/miRDB/policy.html), and mirDIP (http://ophid.utoronto.ca/mirDIP/index.jsp) databases, and then, the candidate differentially expressed mRNAs (DEmRNAs) with targeting binding sites of the target miRNA were obtained from the intersection of DEmRNAs and predicted target genes. GSEA software was used to perform pathway enrichment analysis to study the mechanism of the target miRNA and its target gene involved in BC.

### 2.2. Cell Culture

Human BC cell lines HBL100, 4T1, MDA-MB-231, MDA-MB-361, MDA-MB-435, MDA-MB-468, T47D, and immortalized mammary epithelial cell lines MCF-10A and 184A1 were all obtained from the American Type Culture Collection (ATCC; Manassas, VA, USA). Human BC cell lines were cultured in RPMI 1640 (Invitrogen, Carlsbad, CA, USA) medium containing 10% fetal bovine serum (FBS; Invitrogen, Carlsbad, CA, USA), 100 U/mL penicillin, and 100 *μ*g/mL streptomycin. The MCF-10A cell line was incubated in M-171 medium supplemented with breast epithelial growth factor (Invitrogen, China). The 184A1 cell line was grown in Mammary Epithelial Cell Basal Medium (MEBM; Invitrogen, Carlsbad, CA, USA). All these cell lines were placed in an incubator at 37°C with 5% CO_2_.

### 2.3. Cell Transfection

miR-1258 mimic, E2F1-shRNA, and corresponding negative control (NC) were purchased from GenePharma (Shanghai, China). E2F1-shRNA and E2F1 were subcloned into pcDNA3.1 (Sangon Biotech, Shanghai, China) to construct E2F1 silencing vector (sh-E2F1) and E2F1 overexpression vector (oe-E2F1), respectively. miR-1258 mimic was transfected into cells at a final concentration of 50 nM to overexpress miR-1258. For rescue experiments, miR-1258 mimic or NC mimic was cotransfected with oe-E2F1 or an empty vector (oe-NC) into BC cells. The Lipofectamine 2000 (Invitrogen, Carlsbad, CA, USA) kit was used for all the above transfections. 48 h after transfection, the cells were collected for subsequent experiments.

### 2.4. qRT-PCR

Total RNA was extracted from cells by TRIzol reagent (Life Technologies, Grand Island, NY, USA), and complementary DNA (cDNA) was synthesized using the Reverse Transcription Kit (Applied Biosystems, Foster City, CA, USA) according to the manufacturer's method. The transcription levels of miR-1258 and E2F1 mRNA were determined on the 7500 real-time PCR system using the TaqMan gene expression assay (Applied Biosystems, Foster City, CA, USA). miR-1258 and E2F1 took U6 and GAPDH as internal regulators, respectively. The sequences for the forward and reverse primers synthesized by Sangon (Sangon Biotech, Shanghai, China) were shown in Table 1.

### 2.5. Western Blot (WB)

The transfected cells were seeded at 2 × 10^5^ cells/well in a well plate (Corning, NY, USA). After culture for 72 h, the cells were lysed on ice using RIPA lysis buffer (Beyotime, Shanghai, China) to collect proteins. BCA protein assay kit (Beyotime, Shanghai, China) was used for assessment of the protein concentration according to the instructions. After denaturation at a high temperature, the proteins were separated from a sample loading buffer by sodium dodecyl sulfate polyacrylamide gel electrophoresis (SDS-PAGE) and sequentially transferred onto a polyvinylidene fluoride membrane (PVDF; Millipore, Billerica, MA, USA). Subsequently, the membrane was blocked with 5% skim milk powder for 2 h and then incubated with primary antibodies including rabbit anti-E2F1 (ab179445, 1 : 1000, Abcam, Cambridge, MA, USA) and rabbit anti-GAPDH (ab181602, 1 : 10,000, Abcam, Cambridge, MA, USA) at 4°C overnight, followed by the addition of peroxidase-conjugated secondary antibody (ab6721, 1 : 10000, Abcam, Cambridge, UK) at room temperature for 2 h. Protein signals were detected using an enhanced chemiluminescence kit (GE Healthcare, Chicago, IL, USA).

### 2.6. MTT Assay

Transfected cells were seeded into 96-well plates (Corning, NY, USA) at 5 × 10^3^ cells/well, with a final volume of 200 *μ*L. After 24, 48, 72, and 96 h of incubation, the original medium was replaced with 200 *μ*L of fresh medium, and 25 *μ*M MTT solution (5 g/L^−1^ in phosphate buffer saline) was added to each well to achieve a final concentration of 1 g/L^−1^. The cells were incubated for another 4 h, and then, dimethyl sulfoxide (DMSO; Sigma, St. Louis, MO, USA) was added to dissolve the formed crystal. Detection of the absorbance at 490 nm in the wavelength was carried out with a microplate reader (SpectraMax M2, Molecular Devices, CA, USA).

### 2.7. Colony Formation Assay

Transfected cells were inoculated into 6-well plates (Corning, NY, USA) at 4 × 10^2^ cells/well, with a final volume of 2 mL. The medium was replaced every 4 days. On day 8, cells were fixed with 4% paraformaldehyde (Invitrogen, Carlsbad, CA, USA) and then stained with crystal violet (Invitrogen, Carlsbad, CA, USA) to count the number of stained cells.

### 2.8. Cell Apoptosis Assay

Transfected cells were seeded in a 6-well plate (Corning, NY, USA) with 1 × 10^5^ cells/well for 72 h and then collected by centrifugation at 100 × g for 3 min. After being washed with cold PBS, the cells were stained with FITC-AnnexinV and propidium iodide (PI) according to the instructions of the BD apoptosis assay kit (Becton, Dickinson and Company, Franklin Lakes, NJ, USA) and then analyzed by flow cytometry (FCM, Becton, Dickinson and Company, Franklin Lakes, NJ, USA). Cell QuestPro software (Becton, Dickinson and Company, Franklin Lakes, NJ, USA) was used to analyze FCM data, and the percentage of apoptotic cells was calculated.

### 2.9. Cell Cycle Assay

Transfected cells were seeded in 6-well plates (Corning, NY, USA) at 1 × 10^5^ cells/well for 72 h. After centrifugation at 100 × g for 3 min, the cells were collected and fixed with 70% ethanol at 4°C overnight. After being washed with PBS, the cells were stained with 20 *μ*g/mL PI (Becton, Dickinson and Company, Franklin Lakes, NJ, USA) and 200 *μ*g/mL RNaseA (Becton, Dickinson and Company, Franklin Lakes, NJ, USA) at 37°C for 30 min in dark and immediately analyzed by FCM. ModFit Software (Verity Software House, Topsham, ME, USA) was used to analyze FCM data.

### 2.10. Dual-Luciferase Reporter Gene Assay

Vectors containing wild-type (WT) or mutant (MUT) E2F1 3′-UTR (E2F1-WT, E2F1-MUT) and the control vector pRL-TK (Promega, Madison, Wis. USA) encoding Renilla luciferase were cotransfected with miR-1258 mimic or NC mimic into cells using the Lipofectamine 2000 kit (Invitrogen, Carlsbad, CA, USA). Cells were harvested and lysed 48 h after transfection, and luciferase activity was determined using a Dual-Glo luciferase assay kit (Promega, Madison, WI, USA). The relative ratio of Firefly/Renilla activity was calculated.

### 2.11. Statistical Analysis

All data were processed by SPSS 22.0 statistical software (IBM, SPSS, Chicago, IL, USA), and the measurement data were exhibited as mean ± standard deviation. Differences between two groups were compared by *t*-test, and the differences of more than two groups were analyzed by one-way analysis of variance (ANOVA). The Spearman correlation analysis was used to describe the correlation between miR-1258 and E2F1 expression in BC cells. Each experiment was carried out in triplicate. ^∗^*p* < 0.05 indicated that the difference was significant between groups.

## 3. Results

### 3.1. miR-1258 Is Poorly Expressed in BC Cells

**Figure 1:**
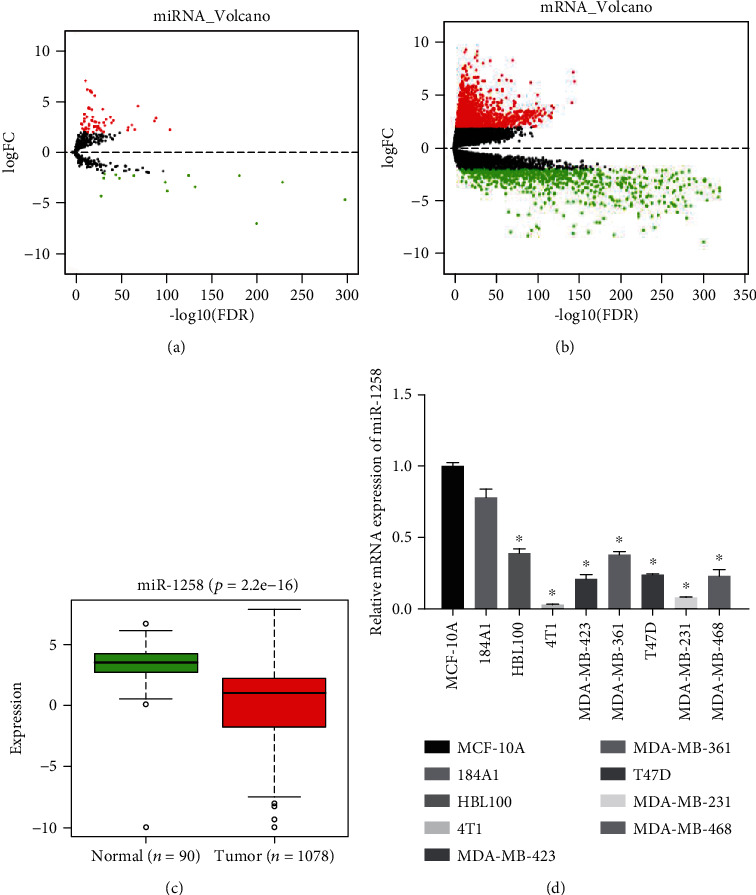
miR-1258 is poorly expressed in BC. (a, b) Volcano plots of DEmiRNAs and DEmRNAs in normal and tumor groups in the TCGA-BRCA dataset. Red dots indicate the upregulated miRNAs and mRNAs in BC, while green dots indicate the downregulated miRNAs and mRNAs. (c) Expression of miR-1258 in the normal and tumor groups in the TCGA-BRCA dataset. (d) Expression of miR-1258 in BC cells and breast epithelial cells. ^∗^ is compared to MCF-10A, *p* < 0.05.

A total of 74 DEmiRNAs and 2,161 DEmRNAs were obtained by differential analysis between BC tumor and normal tissue samples (Figures 1(a) and 1(b)), and miR-1258 was found to be significantly lowly expressed in tumor tissue (Figure 1(c)). Therefore, qRT-PCR was used to detect the expression of miR-1258 in BC cell lines HBL100, 4T1, MDA-MB-435, MDA-MB-361, T47D, MDA-MB-231, MDA-MB-468, and immortalized mammary epithelial cell lines MCF-10A and 184A1 to verify the prediction by bioinformatics. It was observed that miR-1258 was downregulated in all BC cell lines relative to that in MCF-10A and 184A1 cell lines (Figure 1(d)), which was consistent with the bioinformatics result.

### 3.2. Overexpression of miR-1258 Affects Cell Cycle and Proliferation of BC Cells

**Figure 2:**
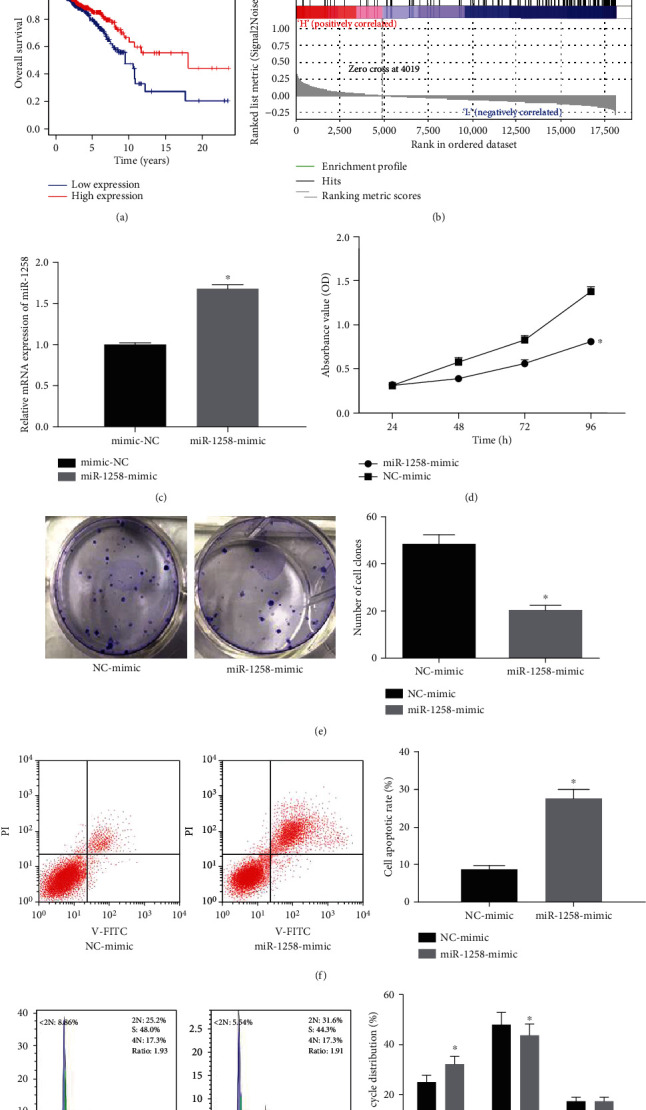
Overexpression of miR-1258 affects the cell cycle and proliferation of BC cells. (a) Survival curves show the prognosis of patients with high expression of miR-1258 (red) and low expression of miR-1258 (blue). (b) GSEA enrichment analysis of miR-1258. (c) Expression of miR-1258 in BC cells after transfection with miR-1258 mimic. (d–g) Effects of miR-1258 overexpression on BC (d) cell proliferation, (e) growth, (f) apoptosis, and (g) cell cycle were detected. The experiments were repeated 3 times, ^∗^*p* < 0.05.

Survival analysis showed that the low expression of miR-1258 in tumor tissue had a significant impact on prognosis, and the survival time of patients with low expression of miR-139 was significantly shorter than those with a high expression (Figure 2(a)). GSEA enrichment analysis exhibited that miR-1258 was closely related to cell cycle (Figure 2(b)). Therefore, the 4T1 cell line with the lowest miR-1258 expression was selected to further study the effect of miR-1258 on BC cell cycle and proliferation. We firstly verified the expression of miR-1258 in the cells transfected with miR-1258 mimic and NC mimic by qRT-PCR, and the result indicated that the content of miR-1258 in the cells transfected with miR-1258 mimic was significantly higher than that in the cells with NC mimic (Figure 2(c)).

After confirming that miR-1258 was overexpressed successfully in cells, MTT (Figure 2(d)) and colony formation (Figure 2(e)) assays were carried out and displayed that the overexpression of miR-1258 reduced the proliferation and growth activity of BC cells. In apoptosis and cell cycle experiments, the overexpression of miR-1258 promoted the apoptosis of BC cells and blocked cells in the G0/G1 phase (Figures 2(f) and 2(g)). In summary, miR-1258 may act as a tumor suppressor to inhibit cell proliferation, thereby suppressing the progression of BC.

### 3.3. E2F1 Is Highly Expressed in BC Cells

**Figure 3:**
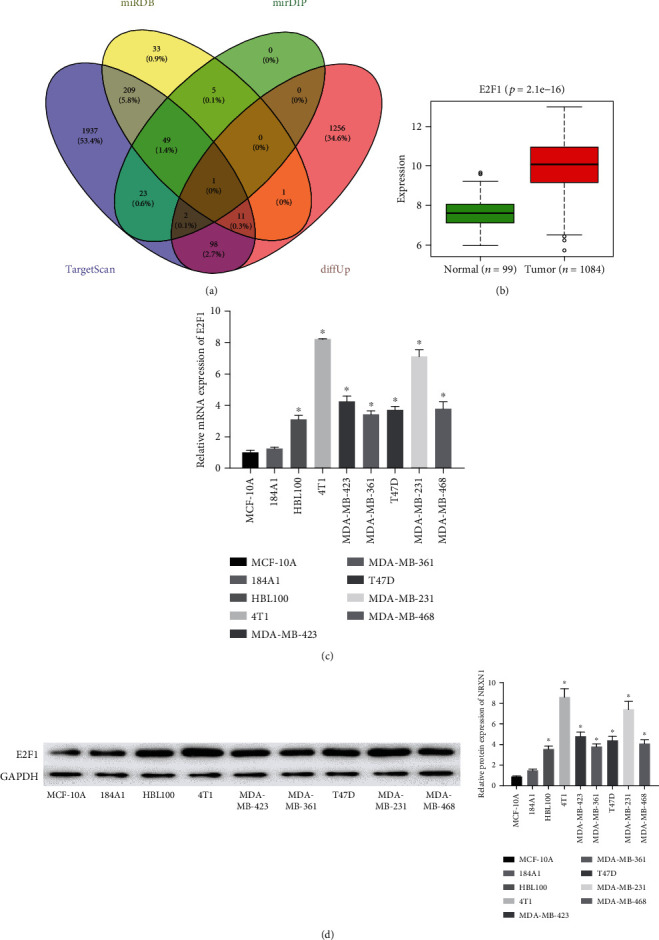
E2F1 is highly expressed in BC cells. (a) Venn diagram shows the intersection between the predicted target genes of miR-1258 and upregulated DEmRNAs. (b) Expression of E2F1 in the normal and tumor groups in the TCGA-BRCA dataset. (c, d) The mRNA and protein expressions of E2F1 in BC cell lines were detected by qRT-PCR and WB. All results were representative of 3 independent experiments, ^∗^*p* < 0.05.

For further study, we predicted the target genes of miR-1258 through TargetScan, miRDB, and mirDIP databases and identified the target gene E2F1 with the binding sites of miR-1258 from the intersection of 1,369 upregulated DEmRNAs and predicted genes (Figure 3(a)). In the meantime, we found that E2F1 was dramatically highly expressed in BC tissue than in normal tissue (Figure 3(b)). Therefore, we further observed the mRNA and protein expressions of E2F1 in BC cell lines and immortalized breast epithelial cell lines through qRT-PCR and WB. The results exhibited that the mRNA and protein expression of E2F1 in BC cell lines were both higher than those in normal cell lines (Figures 3(c) and 3(d)), which was in agreement with the bioinformatics result.

### 3.4. Silencing E2F1 Inhibits BC Cell Cycle and Proliferation

**Figure 4:**
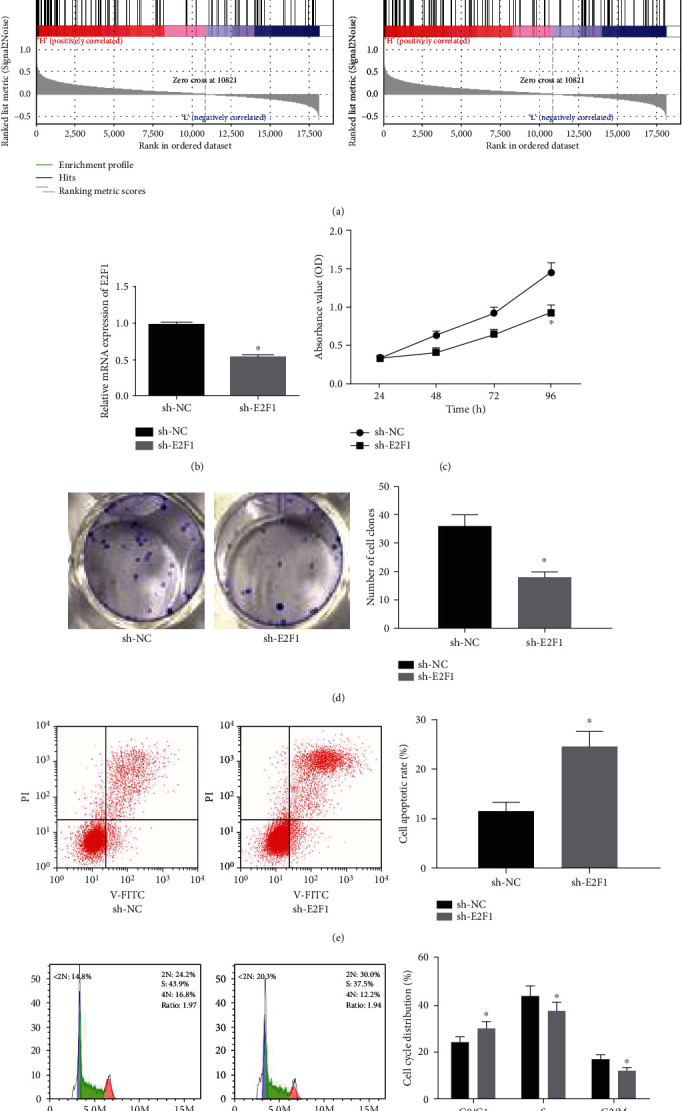
Silencing E2F1 inhibits BC cell cycle and proliferation. (a) GSEA enrichment analysis of E2F1. (b) The mRNA expression of E2F1 in BC cells was tested by qRT-PCR. (c–f) Effects of silencing E2F1 on cell (c) proliferation, (d) growth, (e) apoptosis, and (f) cell cycle were detected by MTT, colony formation assay, and flow cytometry. Each result was a representative of 3 experiments, ^∗^*p* < 0.05.

GSEA enrichment analysis was also conducted on E2F1, and it was found that E2F1 was remarkably enriched in the cell cycle (Figure 4(a)). So, it was necessary to observe the effect of E2F1 on the proliferation and cell cycle of BC cells. At first, we successfully silenced E2F1 in BC cells (Figure 4(b)). Then, MTT (Figure 4(c)) and colony formation (Figure 4(d)) assays were performed, and it was observed that silencing E2F1 inhibited the proliferation and growth of BC cells. In cell apoptosis and cell cycle experiments, silencing E2F1 promoted cell apoptosis and induced cell cycle arrested in the G0/G1 phase (Figures 4(e) and 4(f)), which was in accordance with the results of bioinformatics.

### 3.5. There Is a Targeting Relationship between miR-1258 and E2F1

**Figure 5:**
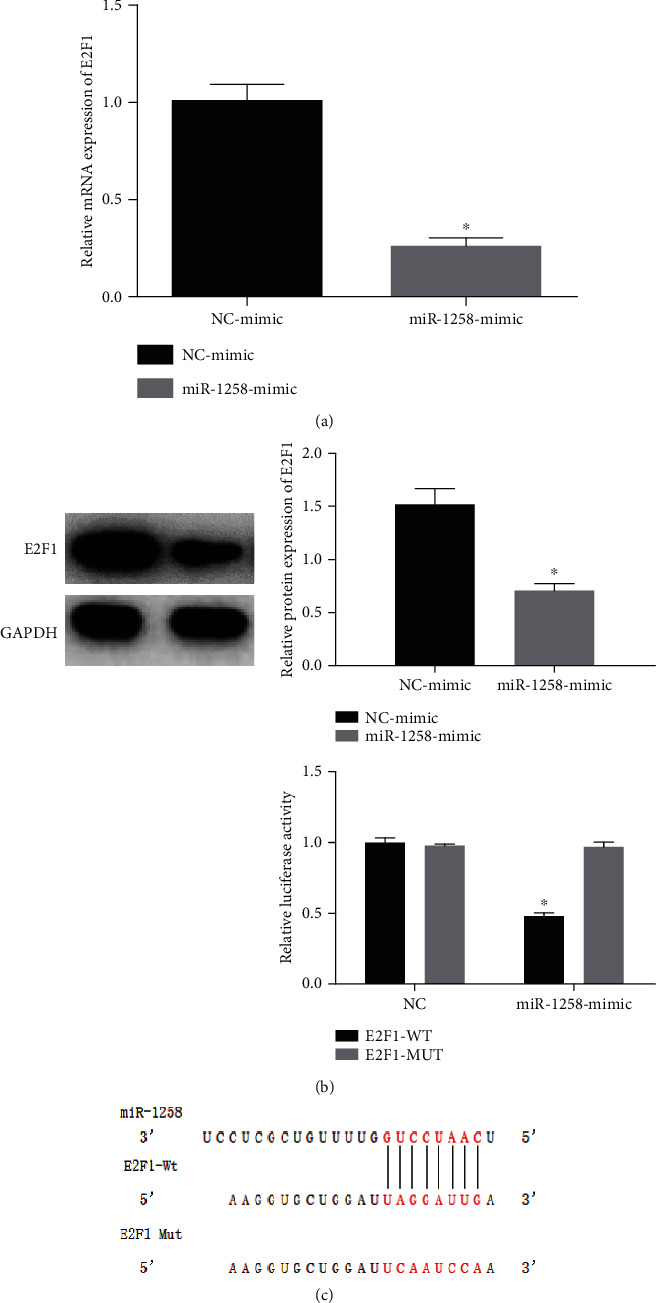
There is a targeting relationship between miR-1258 and E2F1. (a, b) The mRNA and protein expressions of E2F1 after overexpressing miR-1258 in BC cells. (c) The targeting relationship between miR-1258 and E2F1 was validated by dual-luciferase assay. At least 3 independent replicates were performed, ^∗^*p* < 0.05.

Bioinformatics predicted that E2F1 may be the downstream target gene of miR-1258, and the above experiments showed that both of them had effects on the proliferation and cell cycle of BC cells. In order to prove the targeting relationship, qRT-PCR and WB were applied and it was displayed that overexpression of miR-1258 in BC cells significantly decreased the mRNA and protein expressions of E2F1, indicating that miR-1258 could affect the expression of E2F1 (Figures 5(a) and 5(b)). Then, we used dual-luciferase reporter assay to further determine the targeting relationship. The result suggested that compared with the cotransfection group of E2F1-WT and NC mimic, the luciferase activity of the cotransfection group of E2F1-WT and miR-1258 mimic was significantly lower (*P* < 0.05). Meanwhile, the luciferase activity of the cotransfection group of E2F1-MUT and miR-1258 mimic had no significant change relative to that of the group of E2F1-MUT and NC mimic (*p* > 0.05) (Figure 5(c)). The above findings collectively indicated a targeting relationship between miR-1258 and E2F1.

### 3.6. miR-1258 Regulates BC Cell Proliferation, Apoptosis, and Cell Cycle by Targeting E2F1

**Figure 6:**
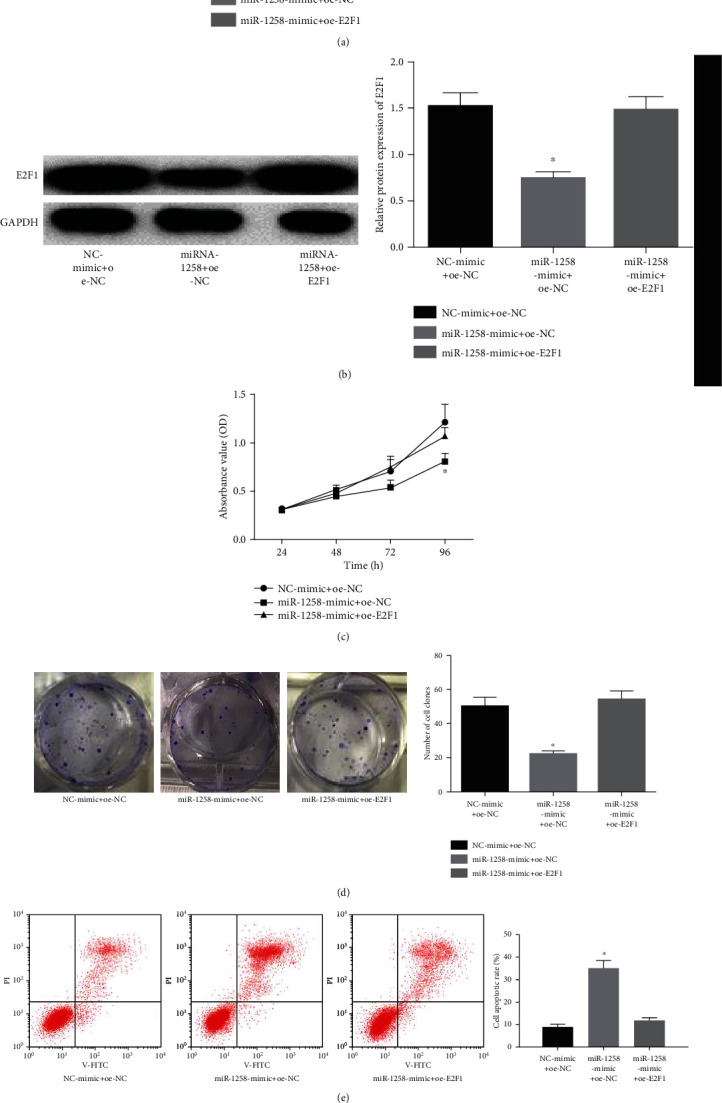
miR-1258 regulates the proliferation, apoptosis, and cell cycle of BC cells by targeting E2F1. BC cells were cotransfected with NC-mimic+oe-NC, miR-1258-mimic+oe-NC, and miR-1258-mimic+oe-E2F1. (a) The mRNA expression of E2F1 was tested by qRT-PCR. (b) The protein expression of E2F1 was tested by WB. (c–f) Effects of miR-1258/E2F1 on BC cell (c) proliferation, (d) growth, (e) apoptosis, and (f) cell cycle were detected by MTT, colony formation, and flow cytometry. The results were representative of at least 3 independent experiments, ^∗^*p* < 0.05.

Through the dual-luciferase assay, we concluded that miR-1258 and E2F1 had a targeting relationship. In order to further confirm the result, we conducted rescue experiments. Three groups were constructed: NC-mimic+oe-NC, miR-1258 mimic+oe-NC, and miR-1258 mimic+oe-E2F1. The mRNA and protein expressions of E2F1 in each group were detected through qRT-PCR and WB, after which MTT, colony formation, and flow cytometry were conducted to assess cell proliferation, colony formation, cell apoptosis, and cell cycle. The results showed that overexpression of E2F1 in BC cells could reverse the inhibiting effect of miR-1258 overexpression on BC cells (Figures 6(a)–6(f)).

## 4. Discussion

BC is a cancer with a high rate of morbidity and mortality, but its pathogenesis remains unclear and there have been no effective curative methods currently [11]. Many previous studies indicated that miRNAs can play an important role in the progression of cancer by directly targeting mRNAs [12, 13]. For instance, miRNAs can act as a tumor suppressor to further inhibit tumor progression through downregulating oncogene translation.

miR-1258 is believed to regulate the cell cycle of multiple tumor cells and inhibit their proliferation. For example, miR-1258 suppresses tumor progression via the GRB2/Ras/Erk pathway in non-small-cell lung cancer [14]. In osteosarcoma, miR-1258 inhibits cell proliferation and promotes cell cycle to be arrested in the G0/G1 phase through targeting AKT3 [15]. Recent studies have discovered that miR-1258 is also associated with BC, and it can inhibit the BC metastasis by targeting heparanase [16]. In this study, we found that the expression of miR-1258 was downregulated in multiple BC cell lines, compared with normal breast cells, and the upregulation of miR-1258 promoted the apoptosis of BC cells, indicating that miR-1258 was a tumor suppressor of BC.

The transcription factor E2F1 family consists of 8 different family members, respectively, which are E2F1 to E2F8. E2F1 as a transcription factor can regulate cell cycle and induce apoptosis of numerous cells. For instance, miR-136 inhibits the proliferation of cervical carcinoma cells by targeting E2F1 and promotes the apoptosis through the NF-*κ*B pathway [17]. CDCA5, transcribed by E2F1, potentiates the initiation of tumor by enhancing cell proliferation and inhibiting apoptosis via the AKT pathway in hepatocellular carcinoma [18]. Relevant literature has reported that E2F1 can promote the progression of BC. For example, E2F1 can drive BC metastasis by changing cell migration via regulating its target gene FGF13 [19]. The long noncoding RNA LINC00511 contributes to BC tumorigenesis by inducing the miR-185-3p/E2F1/Nanog axis [20]. However, the effect of E2F1 on BC cell cycle has not been studied yet. Hence, this study conducted research through relevant experiments and found that E2F1 could induce cell cycle arrest in THE G0/G1 phase and foster the apoptosis of BC cells.

Since both miR-1258 and E2F1 were observed to be able to regulate the progression of BC, we studied their correlation and observed that there is a targeting relationship between miR-1258 and E2F1. In summary, the study demonstrated that overexpression of miR-1258 inhibits BC cell proliferation and blocks cell cycle in the G0/G1 phase, while promoting cell apoptosis via downregulating E2F1. This study provides a basis for the potential of miR-1258 and E2F1 as new indicators for the prognosis and diagnosis of BC and also determines their targeting relationship, which are of profound significance for the study of BC.

## Figures and Tables

**Table 1:** Primer sequences.

Gene	Primer	Sequences
miR-1258	Forward	5′-CTGCGAGTCCCTGGAGTTAG-3′
	Reverse	5′-CGGTGCCCTAACTACCCATT-3′
U6	Forward	5′-CTCGCTTCGGCAGCACA-3′
	Reverse	5′-AACGCTTCACGAATTTGCGT-3′
E2F1	Forward	5′-CCGTGGACTCTTCGGAGAAC-3′
	Reverse	5′-ATCCCACCTACGGTCTCCTC-3′
GAPDH	Forward	5′-GACTCATGACCACAGTCCATGC-3′
	Reverse	5′-AGAGGCAGGGATGATGTTCTG-3′

## Data Availability

The data used to support the findings of this study are included within the article. The data and materials in the current study are available from the corresponding author on reasonable request.

## Additional Points

*Highlights*. The study confirms that miR-1258 is downregulated in breast cancer while E2F1 is upregulated. In breast cancer, miR-1258 can target and downregulate the expression of E2F1. The effects of miR-1258 and E2F1 on the proliferation and cell cycle of breast cancer cells are determined.
